# How do gut feelings feature in tutorial dialogues on diagnostic reasoning in GP traineeship?

**DOI:** 10.1007/s10459-014-9543-3

**Published:** 2014-09-04

**Authors:** C. F. Stolper, M. W. J. Van de Wiel, R. H. M. Hendriks, P. Van Royen, M. A. Van Bokhoven, T. Van der Weijden, G. J. Dinant

**Affiliations:** 1 Department of General Practice, Faculty of Health, Medicine and Life Sciences, CAPHRI School for Public Health and Primary Care, Maastricht University, P.O. Box 616, 6200 MD Maastricht, The Netherlands; 2 Department of Work and Social Psychology, Faculty of Psychology and Neuroscience, Maastricht University, P.O. Box 616, 6200 MD Maastricht, The Netherlands; 3 Department of Primary and Interdisciplinary Care, Faculty of Medicine and Health Sciences, University of Antwerp, Universiteitsplein 1, 2610 Wilrijk, Antwerp Belgium

**Keywords:** Gut feelings, Diagnostic reasoning, GP vocational training, Tutorial dialogues, Intuition, Non-analytical reasoning

## Abstract

Diagnostic reasoning is considered to be based on the interaction between analytical and non-analytical cognitive processes. Gut feelings, a specific form of non-analytical reasoning, play a substantial role in diagnostic reasoning by general practitioners (GPs) and may activate analytical reasoning. In GP traineeships in the Netherlands, trainees mostly see patients alone but regularly consult with their supervisors to discuss patients and problems, receive feedback, and improve their competencies. In the present study, we examined the discussions of supervisors and their trainees about diagnostic reasoning in these so-called tutorial dialogues and how gut feelings feature in these discussions. 17 tutorial dialogues focussing on diagnostic reasoning were video-recorded and transcribed and the protocols were analysed using a detailed bottom-up and iterative content analysis and coding procedure. The dialogues were segmented into quotes. Each quote received a content code and a participant code. The number of words per code was used as a unit of analysis to quantitatively compare the contributions to the dialogues made by supervisors and trainees, and the attention given to different topics. The dialogues were usually analytical reflections on a trainee’s diagnostic reasoning. A hypothetico-deductive strategy was often used, by listing differential diagnoses and discussing what information guided the reasoning process and might confirm or exclude provisional hypotheses. Gut feelings were discussed in seven dialogues. They were used as a tool in diagnostic reasoning, inducing analytical reflection, sometimes on the entire diagnostic reasoning process. The emphasis in these tutorial dialogues was on analytical components of diagnostic reasoning. Discussing gut feelings in tutorial dialogues seems to be a good educational method to familiarize trainees with non-analytical reasoning. Supervisors need specialised knowledge about these aspects of diagnostic reasoning and how to deal with them in medical education.

## Introduction

Diagnostic reasoning is part of the core business of general practitioners (GPs), and teaching diagnostic reasoning has to be a part of GP traineeships. Diagnostic reasoning is generally assumed to be based on the interaction between analytical and non-analytical cognitive processes (Elstein and Schwarz 2002; Hamm 1988; Patel et al. 1999; Norman et al. 2006; Stolper et al. 2011; Boreham 1994), an assumption that has implications for GP training programmes (Eva 2005; Eva et al. 2007). Our study focussed on how diagnostic reasoning was discussed during GP traineeships and how gut feelings as a form of non-analytical diagnostic reasoning featured in these discussions.

Many diagnoses are automatically recognized by experienced GPs (Norman et al. 2006). They immediately interpret a patient’s problem in diagnostic terms and do not engage in elaborate analytical thought processes. The latter are used in more complex patient problems for which the GP has no diagnosis readily available. In both routine and complex cases, gut feelings may automatically arise during the interaction with a patient, and may guide the diagnostic process (Stolper et al. 2009a, 2011). Sometimes a GP becomes aware of a sense of alarm, i.e. the feeling that there may be something wrong with the patient, without knowing exactly what and why. This feeling may activate analytical reasoning in the diagnostic process by stimulating a GP to formulate provisional hypotheses involving potentially serious outcomes. In a similar vein, GPs may perceive a sense of reassurance, i.e. a secure feeling about the further management and course of a patient’s problem, even though they may not be certain about the actual diagnosis (Stolper et al. 2009a, b).

Non-analytical and analytical reasoning processes have been described as two modes of knowing and thinking in dual process theories (Epstein 1994). The non-analytical system is implicit, based on automatic and effortless thought processes, and is associative, intuitive and fast, whereas the analytical system is explicit, controlled, rational, effortful and relatively slow (Epstein 1994; Kahneman and Frederick 2005; Ferreira et al. 2006; Evans and Frankish 2009). During a consultation with a patient, the non-analytical and analytical processes continuously interact and determine the course of the physician’s thinking and actions. The thoughts and feelings activated by the non-analytical system can be reflected upon by the analytical system, and if they are considered useful, analytical strategies such as systematic differential diagnosis, decision tools and causal reasoning about disease processes may be applied(Moulton et al. 2007). Clinical reasoning by experienced clinicians allows fast and efficient diagnoses in complex situations, but may slow down and switch to analytical reasoning when the automatic approach is not enough to explain the patient’s situation or when a sense of alarm arises (Stolper et al. 2011; Moulton et al. 2007). Gut feelings in diagnostic reasoning can be regarded as a specific form of non-analytical reasoning, because of the guiding role of affect defined as a feeling of ‘goodness’ (sense of reassurance) or ‘badness’ (sense of alarm) in the decision process (Finucane et al. 2003; Slovic et al. 2002; Stolper et al. 2011). Most GPs are aware of their gut feelings enabling us to study some aspects of automatic, non-analytical reasoning processes (Stolper et al. 2009a, 2010).

Compared to hypothetico-deductive reasoning, intuitive non-analytical reasoning may lead to better results, i.e. more correct diagnoses (Coderre et al. 2003). Intuitions are often the result of recognizing familiar patterns or the absence thereof, and may be based on only a few relevant signs and symptoms (Klein 2003). In the case of chest pain, gut feelings proved to be rather accurate (Buntinx et al. 1991) and in the case of diagnosing serious infections in children, the family physician’s gut feeling ‘that something is wrong’ proved to be the best predictor among all signs and symptoms (Van den Bruel et al. 2007, 2010). The gut feeling that ‘this is not normal’, which may arise when a physician observes a child, has proved to be a sign that makes the physician question the child’s well-being (Lykke et al. 2008). Other researchers found that a suspicion of cancer was sometimes based on intuitive knowledge, causing a GP to become concerned (Johansen et al. 2012). The sense of alarm as a diagnostic tool has been taken seriously by disciplinary tribunals and is even regarded as an element of the professional standards for doctors (Stolper et al. 2010a).

Although the substantial contribution of non-analytic reasoning to the diagnostic process is obvious, it is a topic of debate whether this kind of reasoning can be taught. Some authors have argued that intuitive reasoning can be enhanced and improved (Klein 2009; Kahneman and Klein 2009; Hogarth 2001, 2010). Research has shown that GP supervisors discuss the role of gut feelings in diagnostic reasoning with their trainees (Stolper et al. 2009a) and that instructions to use a combination of analytical and non-analytical reasoning may improve students’ diagnostic accuracy (Eva et al. 2007; Ark et al. 2007). There is also some evidence that taking time for personal reflection and getting immediate feedback may improve the quality of the diagnostic reasoning process and may reduce cognitive errors (Mamede et al. 2008, 2010, 2012; Coderre et al. 2010; Elstein 2009; Graber et al. 2012; Ericsson 2004). It is unknown whether these different approaches are applied in GP traineeships.

GP traineeships in the Netherlands take 3 years, and trainees spend their first and third years working in a general practice, where they mostly see patients alone and consult with their supervisors on a regular basis to discuss patients and problems, receive feedback and improve their competencies. We refer to these face-to-face meetings with the supervising GP as tutorial dialogues (TDs) (Chi et al. 2008; Vanlehn et al. 2007). It is unknown how diagnostic reasoning is discussed in these tutorial dialogues and whether gut feelings play a role. Our first research question therefore was what supervisors and their GP trainees discuss in tutorial dialogues on diagnostic reasoning. The second question was how gut feelings feature in these dialogues. Therefore, we used both qualitative and quantitative methods to describe the topics discussed, the nature of diagnostic reasoning, and how and to what extent gut feelings feature in these dialogues.

## Methods

### Data collection

We approached (2010) all eight Departments of General Practice of universities in the Netherlands, inviting them to collect video recordings of TDs on diagnostic reasoning in general, thus without disclosing our second research question We received 21 TDs from seven departments. Four of them did not fit the inclusion criteria (recording too poor, participants unknown or dialogue not related to diagnostic reasoning about patients). The participants were 16 GPs (6 female) and 16 trainees (14 female). We received two recordings from the same supervisor and trainee. Five trainees were in their first year of training and 12 in their third year. The participants were from all over the country. The current percentage of female GP supervisors in the Netherlands is about 30 %, and the percentage of female trainees is about 75 % (source: SBOH, the employer of GP trainees, www.sboh.nl, August 2012). The gender distribution in our sample (36 % female supervisors and 86 % female GP trainees) differed little from these national data. In one TD a dietician shortly entered in the middle of the conversation but had an insignificant contribution to the conversation.

### Data analysis

The 17 TDs were transcribed. Both a qualitative and a quantitative analysis was performed. The aim and content of the TDs were described and the diagnostic reasoning process was analysed. Illustrative quotes were selected. Atlas.ti was used for a detailed bottom-up and iterative content analysis and coding procedure (Neuendorf 2002; Krippendorff 2012). At least one code was assigned to each turn in the dialogues; turns were further segmented into quotes based on content changes. After 5 protocols had been analysed, a coding scheme was drawn up by a team of four researchers. All authors were informed about the procedure and the progress via interim reports, and discussed them in regular meetings. Two researchers divided up all protocols between them for coding, checked each other’s codes, and discussed the differences until agreement was reached. After 13 transcripts had been coded, the coding scheme appeared to be complete: no new codes were necessary and saturation was reached.

Table 1 presents the main categories in our coding scheme. We distinguished between a reporting phase in which the trainee reported his/her actual diagnostic reasoning during the patient consultation, and an analysis phase in which both the supervisor and the trainee analysed the process of diagnostic reasoning. In both the reporting and analysis phase, five main categories related to topics of discussion were distinguished: diagnostic reasoning (DR), management, communication, diagnostic reasoning process, and gut feelings. In addition, two other categories described the quotes that were used to structure the TDs or that were irrelevant. The whole text was coded, quotes did not overlap and each quote was given a content code referring to the topic of discussion and a speaker code referring to either the supervisor or the trainee. This procedure enabled us to use the number of words per code as a unit of analysis to describe how much attention was paid to certain topics as well as to compare the contributions made by trainees and supervisors. (Chi 1997)

**Table 1:** Mean number of words used in a tutorial dialogue and the percentage of words per coding category for trainees and supervisors

Category (N codes)	Mean number of words per TD (N = 17)			Percentage of words per TD (N = 17)		
	Trainee	Supervisor	Total	Trainee	Supervisor	Total
*Reporting phase*						
DR (33)	636.9	138.5	775.4	13.9	3.1	17.0
Management (7)	122.3	77.3	199.6	2.6	1.8	4.4
Communication (1)	70.0	5.1	75.1	1.5	0.1	1.6
DR Process (3)	10.2	8.4	18.6	0.2	0.3	0.5
Gut feelings (11)	0.7	5.6	6.3	0.0	0.2	0.2
*Analysis phase*						
DR analysis (33)	1,217.1	1,092.2	2,309,3	25.5	23.5	49.0
Management analysis (5)	287.4	256.2	543.6	6.0	5.8	11.8
Communication analysis (1)	79.2	69.7	148.9	1.7	1.3	3.0
DR process analysis (3)	7.7	0.0	7.7	0.2	0.0	0.2
Gut feelings analysis (11)	42.8	57.2	100.0	1.3	1.6	2.9
Structuring the TD (11)	117.0	163.4	280.4	2.5	3.8	6.3
Unspecified (2)	64.5	69.1	133.6	1.4	1.6	3.0
Total	2,655.8	1,942.7	4,598.5	56.8	43.1	99.9 %

In 1 TD a dietician shortly entered in the middle of the conversation and had an insignificant contribution **(**TD9: 207 words, 0.1 % total**)**. Therefore, the total percentage of words in the table is only 99.9 %. All mean numbers represent weighted average values per case

## Results

### Description of TDs

**Fig. 1:**
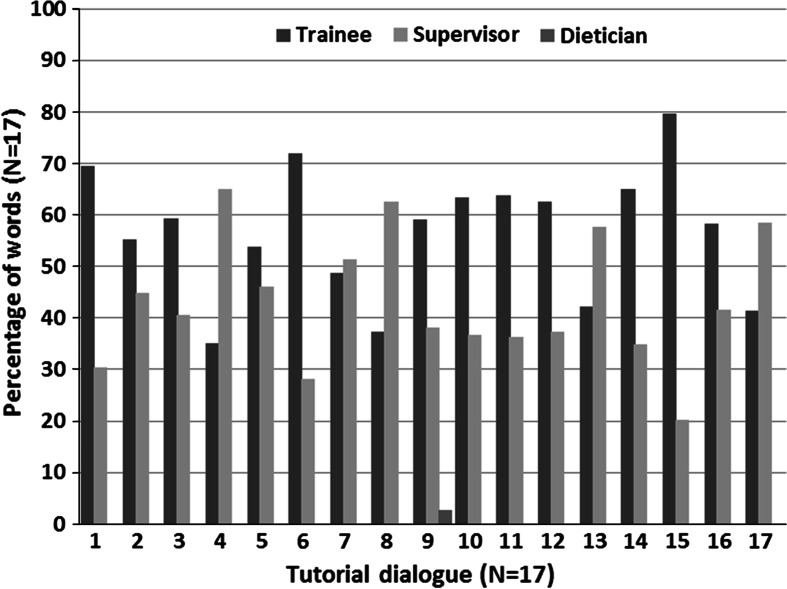
Percentage of words per tutorial dialogue for each participant (supervisor, trainee, dietician)

Most of the TDs were debriefings about one or more patients encountered by the trainees during the office hours (n = 13). In the 17 TDs, 44 patient cases were discussed (mean 2.6, range 0–7), 37 initiated by the trainees and 7 by the supervisors. The dialogues lasted on average of 32.5 min (range 13–57). These meetings often had a rather routine character and the aim, i.e. debriefing patients, was mostly not explicitly stated. On some occasions (n = 4) a trainee formulated a clear goal such as seeking advice for a differential diagnosis, presenting the results of a literature search on the significance of a diagnostic test or discussing the diagnostic role of gut feelings. In TD number 16, the supervisor presented his own diagnostic problem with a patient and asked the trainee to think along with him. In TD number 17, the supervisor put a lot of effort into instructing the trainee in the use of cross tabulation (see Fig. 1). In another case, a supervisor asked the trainee for an account of his reasoning process based on a patient’s record. Only two cases involved an evaluation of the TD, i.e. what the trainee got out of it, or an appointment for follow-up. In general, the discussion ended when there were no more patients to be discussed. Even the discussions about patients were often open-ended, i.e. without conclusions or agreements (32 of 44 cases).

### Topics discussed

Table 1 shows that most words were used for reporting diagnostic reasoning (DR) and analysis of diagnostic reasoning (ADR) (17.1 and 49.1 % respectively), while 3.1 % of the words concerned gut feelings. Overall, trainees made a larger contribution in terms of the numbers of words during the TDs than their supervisors (with mean shares of 56.8 and 43 % of words respectively), but especially in the reporting phase. In the analysis phase, supervisors and trainees contributed almost equally to the discussion. However, some supervisors talked much more than their trainees (see Fig. 1, TD numbers 4, 8, 13 and 17), especially in the analysis phase. Table 2 shows how the main codes are distributed per category, what percentages of words per code were found in the reporting and analysis phases and in how many of the TDs (the complete table can be found on www.gutfeelingsingeneralpractice.eu). Table 2 presents the main elements discussed during the dialogues and their importance in terms of the numbers of words used for the topic and the frequency of occurrence. Most TDs started with the trainee’s presentation of a patient, and Table 2 shows that this presentation had a prominent place in the reporting phase (17.1 % in 16 TDs). Other elements of diagnostic reasoning, such as history-taking and physical examination, were important topics in both the reporting (1.6 % in 14 TDs and 2.4 % in 14 TDs respectively) and analysing phases (2.3 % in 15 TDs and 2.3 % in 14 TDs respectively). In their analysis of diagnostic reasoning, supervisors focussed on listing differential diagnoses (8.2 % in 17 TDs), asking trainees to explicitly indicate what information in the case history guided their reasoning process (3.3 % in 15 TDs), and what data might confirm or exclude diagnostic options (6.5 % in 17 TDs). Regarding knowledge, several sources of knowledge played a major role in this analysis of reasoning. Contextual knowledge, defined as everything a physician knows about his/her patient apart from the signs and symptoms, (Hobus et al. 1987) was discussed in both the reporting and analysis phases (0.9 % in 15 TDs and 2.3 % in 15 TDs respectively). Medical knowledge, including disease-specific, epidemiological and therapeutic knowledge, as well as experiential knowledge, was most used in the analysis phase (7.9 % in 17 TDs and 1.9 % in 14 TDs respectively). In addition, patient management was frequently discussed in our sample, particularly in the analysis phase (11.8 % in 17 TDs) and often in relation to the proposed diagnoses. Other topics, such as communication skills or organizational problems, were also discussed.

**Table 2:** Mean percentage (rounded to one decimal) of words used in a tutorial dialogue for main codes in the coding categories diagnostic reasoning, gut feelings, diagnostic reasoning process, management and communication in both the reporting and analysis phase of diagnostic reasoning and number of TDs in which they occurred

	Phase of reporting of diagnostic reasoning		Phase of analysis of diagnostic reasoning		Total	
	% words (range)	In N TDs	% words (range)	In N TDs	% words	In N TDs
*Diagnostic reasoning*	17.1 (0.0–35.7)	16	49.1 (22.5–75.4)	17	66.2	17
Presentation of patient	8.2 (0–23.4)	14	1.1 (0–10.7)	6	9.3	15
Differential diagnosis	0.1 (0–1.0)	2	8.2 (0.6–29.7)	17	8.3	17
Medical/epidemiological/therapeutic knowledge	0.2 (0–1.1)	6	7.9 (0.4–56.7)	17	8.1	17
Making diagnostic considerations more explicitly	0.3 (0–1.6)	6	6.5 (0.5–19.3)	17	6.8	17
Making relevant history more explicitly	1.4 (0–4.8)	13	3.3 (0–7.9)	15	4.7	16
Physical examination	2.4 (0–8.6)	14	2.3 (0–8.2)	14	4.7	15
History-taking	1.6 (0–3.7)	14	2.3 (0–8.8)	15	3.9	16
Contextual information	0.9 (0–4.1)	11	2.3 (0–8.6)	15	3.2	15
Experiential knowledge	0.4 (0–3.0)	6	1.9 (0–10.4)	14	2.3	14
*Gut feelings*	0.2 (0–1.2)	4	2.9 (0–43.6)	6	3.1	7
Significance	0.1 (0–0.8)	3	0.4 (0–4.0)	5	0.5	5
Process	0.0 (0–0.3)	1	0.5 (0–7.8)	2	0.5	3
Description	0.0	0	0.4 (0–7.1)	2	0.4	2
Triggers, cues	0.0 (0–0.6)	1	0.4 (0–7.1)	2	0.4	3
Learning process	0.0	0	0.4 (0–6.2)	1	0.4	1
Validity	0.0	0	0.3 (0–4.4)	1	0.3	1
Determinants	0.0	0	0.2 (0–3.4)	2	0.2	2
Example	0.1 (0–0.9)	1	0.2 (0–2.8)	1	0.2	2
GP traineeship	0.0	0	0.1 (0–0.9)	1	0.1	1
Out-of-office hours	0.0	0	0.1 (0–0.8)	1	0.1	1
Shared decision-making	0.0	0	0.0 (0–0.5)	1	0.0	1
Diagnostic reasoning process	0.5 (0–5.9)	7	0.2 (0–1.5)	*4*	0.7	10
Management	4.4 (0–9.6)	16	11.8 (4.4–30.4)	17	16.2	17
Communication	1.6 (0–7.4)	11	3.0 (0–14.7)	10	4.6	15

### Diagnostic reasoning

The TDs in our sample generally consisted of analytical reflections on the trainees’ diagnostic reasoning during their consultations. The trainees presented a retrospective reconstruction of their thinking as a rather rational process of testing plausible hypotheses, with little attention for the role of associative reasoning. In our sample, the participants never took a bird’s-eye view of this reconstructing process to become aware of possible tacit clues or intuitive hunches hidden in the patient’s story or context.

The TDs showed clear evidence of hypothetico-deductive reasoning, with an emphasis on explicitly evaluating relevant information and testing provisional hypotheses: what is the differential diagnosis, what questions can be asked, what physical examination can be done and what further testing (laboratory tests, X-rays, etc.) must be ordered to exclude or confirm a provisional diagnosis. Supervisors stimulated trainees to use this deductive form of reasoning.

- Which complaints don’t fit in with a trigger finger? (TD15, supervisor) The fact that they subside within 15 min. So that’s all a bit atypical. (TD15, trainee) So that may be something to put somewhere at the lower end of the differential diagnosis. (TD15, supervisor) But I thought it didn’t quite fit, but on the other hand the complaints that she only has in the morning do fit in, but then the fact that it subsides and she has no complaints for the rest of the day, that’s unexpected. (TD15, trainee) (quotation a)

The question when a GP trainee might assume his/her list of hypotheses to be sufficiently comprehensive was never asked. One trainee explained his diagnostic strategy of doing a complete history and physical exam. The guidelines (‘Standards’) of the Dutch College of General Practitioners and other guidelines played a minor part and computerized decision support systems were never used. Another trainee mentioned that the simultaneous combination of communicating and diagnostic reasoning was difficult.

- What I can remember … from this consultation … I was working very hard … to get a grip on it. As the complaints are rather vague. What I can’t immediately come up with is what we’re going to do about it. Because er …. What’s uppermost in your mind? Or in technical terms what is the differential diagnosis… But indeed I don’t know what she wants herself. (TD12, trainee) You do ask relevant questions, but you’re not asking about the patient’s own request for help. You’re not asking about the context. You don’t know what’s in her head … Also I never heard you ask any questions about her feelings. (TD12, supervisor) All I’m asking myself is what on earth am I going to do with this patient? (TD12, trainee) Exactly, and so you’re skipping certain steps that are probably very important in the case of this patient. (TD12, supervisor) Right. And which would probably have given me more useful information than all those diagnostic options that kept buzzing around in my head. (TD12, trainee) (quotation b)

Apart from gut feelings, reference to non-analytical reasoning was identified only in two TDs.

- Are there any other ideas, options [in terms of differential diagnostics]? (TD5, supervisor) Yes, a brainstem haemorrhage. That was another possibility I was thinking about. (TD5, trainee) Why specifically the brain stem? (TD5, supervisor) Because I’d had a patient with complaints in one arm, and that was in the brain stem. So that’s what automatically came to mind. But I don’t know whether it could be explained by a problem elsewhere in the brain. (TD5, trainee) (quotation c)

No one reported an instantaneous diagnosis based on sudden recognition of a pattern. Sometimes supervisors asked questions that were related to trainees’ non-analytical reasoning, stimulating them to be aware of the way their assessments of patients had come about.

- You said: ‘When I entered the room she was lying in bed, and was obviously not as fit as usually. But she didn’t impress me as being very ill’. What did you watch out for? What caused her not to appear very ill to you? (TD1, supervisor) (quotations d)
- Mmm, yes … you were worried about certain aspects. What were the aspects that triggered this concern? (TD4, supervisor) (quotation e)
- [In response to a question by the supervisor about the assessment of a mammary tumor:] It was so tender to the touch that it made me think it could hardly be a tumour, and it felt very smooth. (TD3, trainee) It’s the kind of thing you tend to do intuitively. (TD3, trainee) But I think that’s important, what you’re saying, that you … how the tumor felt to you, that it’s smooth, so that apparently guided you towards …, probably a cyst. (TD3, supervisor) (quotation f)
- The key could be to put it into words as much as you can if you get this feeling of, like, you think it’s your intuition, so you can see if you could specify it in concrete terms. Whether you can say all right … but what’s this intuition based on? (TD2, supervisor) (quotation g)

### Gut feelings

Gut feelings or descriptions of gut feelings were discussed in 7 TDs. These discussions were initiated both by supervisors and trainees, most often when talking about diagnostic uncertainty (n = 5). Table 2 shows that 3.1 % of the number of words in the dialogues concerned gut feelings, and Table 1 shows that supervisors contributed more to this topic than trainees. In TD number 2 a trainee started a discussion about the significance of gut feelings in diagnostic reasoning, which took up 33.4 % of the total number of words in this TD. Table 2 shows that the significance of gut feelings in diagnostic reasoning (0.5 %), the process of developing gut feelings (0.5 %), the description of gut feelings (0.4 %), the triggers and cues inducing gut feelings (0.4 %) and the learning process (0.4 %) were most frequently discussed.

Gut feelings were considered to play a functional role in a dynamic reasoning process, and supervisors tried to explain how this may work. Both supervisors and trainees described the gut feelings phenomenon and discussed questions including: what is a gut feeling, when did a gut feeling arise, how can this process be explained, when can you trust your gut feeling, what aspect of a patient’s presentation made a gut feeling arise and what was the role of gut feelings in the diagnostic reasoning and management process (see Text box 1).

**Text box 1:** Selection of quotations of supervisors and trainees with examples of gut feelings

*Description of gut feelings* • It’s a particular feeling that you get, and that you then try to confirm with facts … In my view, gut feelings mean that you make a distinction, you have two people with identical symptoms, and you still think, with one of them, this is suspicious, it’s going to develop into such and such, and with the other you think, it’s OK to wait and see… I’m fascinated to know what exactly that is, and what makes you make a distinction and choose a particular direction. (TD2, trainee) (quotation h) *Arising of gut feelings* • Is this something that also involves your gut feeling? … That you sometimes think this just doesn’t fit? That it worries you? I mean if someone’s just had a heavy cold, or is known to suffer from Meniere’s disease, or err… that sort of thing, then you’re easier in your mind than if someone gets this kind of acute attack out of the blue. (TD13, supervisor) (quotation i) *Explanation of process leading to gut feelings* • It’s probably a whole mishmash of information, and that doesn’t immediately fit into a formal flow chart but in your associative memory it sort of tends towards a particular direction, your thoughts move in certain direction so that that is the sense of alarm or reassurance. It’s something that you can start to trust more and more as you become more experienced … I think it relates to having information at several levels and absorbing it at that moment with that specific patient and based on your own experience, perhaps not specifically with this patient but what you have seen with other patients. (TD2, supervisor) (quotation j) *When to trust gut feelings* • Of course it’s also a matter of gaining experience and continuing to test your hypotheses against the outcome to see whether … how it relates to your sense of alarm or sense of reassurance. (TD2, trainee) (quotation k) • See also quotation j above. *Patient information that triggers gut feelings* • It’s sometimes very subtle. I mean you’ve got someone whose story makes you think it could be someone with an appendicitis, you examine her, you think, well it could be, but I’m not sure. And then the patient gets up off the examination table and she walks off in a certain way that makes you think I’m going to refer her anyway. And that’s got to do with, well, … her way of walking at that moment … and then you’ve checked it all and it all fits in … So it’s, well, a combination of specific things that you can actually check off, and a kind of general feeling … I guess. (TD2, supervisor) (quotation l) • Actually up until the physical examination I was still thinking it’s not so serious. And then you also left, and you also seemed to think well … it’s an obvious case. And then it turned out when I examined … that she had more complaints than I’d expected. And so I started to ask some other questions, and found some more information … and then I thought, actually, that doesn’t really fit in at all, with kidney stones… And then I thought there might be something else than just kidney stones. It was kind of an unusual story for just kidney stones. (TD4, trainee) (quotation m) • But it was a lady who really had rather an unusual story for someone who’s basically very healthy. (TD12, trainee) You haven’t got a diagnosis, but you have a sense of alarm about this woman, at this age, who’s always in good health. Who doesn’t ever really consult her GP. (TD12, supervisor) (quotation n) • See also the quotations f and g in the main text *Role of gut feelings in the diagnostic reasoning and management process* • And … errm… you’re saying suppose it all turns out negative… we could of course still decide to wait and see. But I think that wouldn’t quite reassure you. You’d tend to ask a gynaecologist. (TD14, supervisor) (quotation o) • But this diagnostic reasoning. How else could we have, sort of, approached that? (TD4, trainee) I don’t know that it actually went wrong. It’s more that you should be aware of what process [of diagnostic reasoning] are we dealing with? What kind of process are we trying to shape? And then if you look into it, there’s a good chance that it might improve. So just take some time to analyse. Where did that uneasy feeling come from? What makes you need my opinion? (TD4, supervisor) (quotation p) • The only reason why I thought perhaps it should be done sooner, could it be a retrocoecal appendicitis. That was my argumentation, that, well, more should be done. I’ve now referred her for an ultrasound. … But, err, I find it a difficult decision, so that’s why I said, well, if someone develops a fever then it should be evaluated immediately by a surgeon, but that fever had also subsided. (TD16, supervisor) (quotation q) • See also quotation h in the main text	

The number provided in brackets after each turn refers to the TD number

In 6 TDs, the sense of alarm appeared to be a common concept that was considered a useful tool in diagnostic reasoning. Discussing gut feelings led to reflection on the actual diagnostic reasoning process (TDs numbers 2, 3, 4 and 16), and in some cases made a trainee aware of clues hidden in a patient’s story and context (TD numbers 2 and 3). One supervisor explained gut feelings as a vital element since they may guide the reasoning process when a physician is standing at a crossroads where various options, such as watchful waiting or intervening, are still open. This supervisor assured his trainee that experience enables you to trust your gut feelings more (see Text box 1 quotation j). One trainee suggested that asking for feedback on the outcome of a gut feeling may enhance the learning process. Another supervisor argued that it is not important whether a sense of alarm is correct or incorrect, but that it has to make a trainee aware of the reasoning process itself, making them slow down by taking time to analyse where this uneasy feeling comes from (see Text box 1 quotation p). One trainee explained that a sense of alarm may arise because patterns and provisional hypotheses no longer fit (see Text box 1 quotation m). A sense of alarm might be a reason to consult the supervisor(see Text box 1 quotation p) or to formulate provisional hypotheses involving potentially serious outcomes, and to arrange further testing sooner than usual (see Text box 1 quotation q). One supervisor asked what course of action the trainee would choose when this uneasy feeling would still persist even after diagnostic testing (see Text box 1 quotation o). One dialogue featured an example of a sense of reassurance (see above quotation f). Knowledge about a patient’s history and experience with patients in general were regarded as important determinants (see Text box 1 quotations j and n). One trainee told her supervisor that the contextual information had made her more alert (see Text box 1 quotation n). And in one TD, previous experiences appeared to wrongly reassure a trainee when he explained that a smooth and sore mammary tumour is unlikely to be malignant (see above quotation f) .

## Discussion

The purpose of our study was to explore the discussions about diagnostic reasoning in tutorial dialogues of supervising GPs and their trainees and how gut feelings featured in these dialogues. A major finding in our study is that gut feelings, a specific form of non-analytic diagnostic reasoning, were discussed in 7 of the 17 TDs, sometimes playing a small role and sometimes a substantial one. In those TDs gut feelings were regarded as a useful tool in diagnostic reasoning, specifically in situations of diagnostic uncertainty. Discussing gut feelings enhanced the awareness of non-analytical aspects of diagnostic reasoning. When diagnostic reasoning was discussed more generally, this was sometimes prompted by the functional role played by gut feelings in the process. Some supervisors explained to the trainees that the sense of alarm needed to activate diagnostic thinking, i.e. analytic reasoning, by stimulating them to reconsider provisional hypotheses and to look for cues responsible for the uneasy feeling. Other automatic reasoning processes, such as pattern recognition and the automatic generation of hypotheses, received less attention, whereas analytical reasoning played a leading part.

The main topic in the 17 tutorial dialogues on diagnostic reasoning concerned the debriefing of patients. Trainees reported their diagnostic reasoning during encounters with patients and both supervisors and trainees analysed this reasoning process, often by hypothetico-deductive reasoning using several knowledge sources. The aim of the dialogues was mostly not explicitly formulated, evaluation was quite often lacking, and the discussion of patients was usually open-ended. Remarkably, guidelines hardly featured in our TDs. Although the literature about guidelines mostly regards therapeutic interventions, (Davey et al. 2011) while our TDs focussed on diagnostic reasoning, 16.2 % of the words in our sample were spent on patient management, so we would have expected guidelines to be discussed in some TDs.

Results of previous research into gut feelings in general practice (Stolper 2010) enabled us to triangulate. These results showed that experienced GPs recorded a sense of alarm in consultations with 7.4 % of all patients seen during office hours, and inexperienced GPs in 11.4 %, figures which were doubled during out-of-office hours (Stolper 2010). The sense of alarm (n = 6) seems to be sufficiently represented in our TDs. A sense of reassurance, however, was mentioned in only one TD. Many TDs only discussed patients with diagnostic problems and in most cases it was the trainee and not the supervisor who decided which patients needed to be discussed and why. Apparently, the supervisors assumed that the diagnostic problems posed by the other patients seen by the trainee had been correctly solved, which might be a questionable assumption. This may explain why so little attention was paid to the contribution of a sense of reassurance, which might be an educational shortcoming.

As regards the internal validity of our study, the sample of 17 TDs on diagnostic reasoning seems to be representative as we included TDS from different supervisors and trainees related from different vocational training institutes. The high percentage of words relating to diagnostic reasoning (66.2 %) showed that our inclusion process had its intended result. However, acquiring 21 TDs took a great deal of time. Many supervisors promised us to send a recording of a TD but in fact did not. Reasons were lack of time and lack of appropriate recording devices. Some supervisors said that TDs specifically focussing on diagnostic reasoning were unusual. It is possible that discussions about gut feelings also occur in TDs not focussing on diagnostic reasoning, e.g. those discussing management aspects, so these may have been missed.

We did reach data saturation, since we did not need any new codes after coding 13 transcripts. Our 17 TDs, involving 16 different supervisors and their trainees, delivered 9.2 h of dialogue, and showed a large and rich variety of data. Selection bias for favouring aspects of non-analytical reasoning is unlikely, as we asked the supervisors to provide TDs about diagnostic reasoning in general. We found that gut feelings were discussed in 7 of the 17 TDs (41 %); it may be questioned whether this is representative but this was not specifically aimed for.

Our combined qualitative and quantitative approaches proved useful, since the results of the quantitative analysis of the coding categories clearly described what topics were discussed most in TDs on diagnostic reasoning and by whom. This visualized the role of gut feelings in diagnostic reasoning and its analysis. The quantitative descriptions provided a good basis to select topics for further qualitative descriptions.

As for the external validity of our study, GPs in the Netherlands, frequently use a typically Dutch expression (*pluis/niet*-*pluis*) to indicate gut feelings (Stolper et al. 2009a) and we assume that familiarity with this concept facilitates discussions about gut feelings among Dutch GPs. The sense of alarm, however, is a familiar phenomenon in general practice all over Europe and is also often referred to by specific phrases in different languages (Stolper et al. 2010b). We think therefore that our results are of vital importance for GP vocational training institutes in other countries where TDs are a common learning tool in workplace settings.

TDs addressing diagnostic reasoning are by nature retrospective and analytical. Discussing gut feelings may help to discern non-analytical elements in diagnostic reasoning and their value in this process. Similarly, when discussing problems encountered in the diagnostic process, (Van de Wiel et al. 2011; Slotnick 1996, 1999) discussions of gut feelings may improve trainees’ diagnostic competence. Specialised knowledge about these often hidden facets of diagnostic reasoning, including how to deal with them in medical education and TDs, is important for supervisors, and we recommend including these aspects as a substantial component in training-the-trainers programmes. To our knowledge this study is one of the first studies that analysed the main topics of discussing diagnostic reasoning in tutorial dialogues although tutorial dialogues are very common in educational settings in medicine. Diagnostic reasoning belongs to the heart of general practice and research on the learning of this process in workplace settings is needed. Future research into the dialogues between supervisors and trainees may result in more effectiveness of trainees’ learning processes.
